# Catheter-related blood stream infections in hemodialysis patients: a prospective cohort study

**DOI:** 10.1186/s12882-017-0773-5

**Published:** 2017-12-08

**Authors:** Stephanie Thompson, Natasha Wiebe, Scott Klarenbach, Rick Pelletier, Brenda R. Hemmelgarn, John S. Gill, Braden J. Manns, Marcello Tonelli, for the Alberta Kidney Disease Network

**Affiliations:** 1 Division of Nephrology and Immunology 11-112 CSB, 152 University Campus NW, University of Alberta, Edmonton, AB T6G 2G3 Canada; 2 Department of Renewable Resources, Faculty of Agriculture, Life and Environmental Sciences, University of Alberta, 442 Earth Sciences Building, Edmonton, AB T6G 2E3 Canada; 3 Division of Nephrology, University of Calgary, Foothills Medical Centre, 1403-29th Street NW, Calgary, AB T2N 2T9 Canada; 4 Division of Nephrology and Centre for Health Evaluation and Outcome Sciences, University of British Columbia, BC 1081 Burrard Street Vancouver, Vancouver, BC V6Z 1Y6 Canada; 5 Department of Medicine, University of Calgary, 7th Floor, TRW Building, 3280 Hospital Drive NW, Calgary, AB T2N 4Z6 Canada

**Keywords:** Hemodialysis, Bacteremia, Catheter, Residence location

## Abstract

**Background:**

For people requiring hemodialysis, infectious mortality is independently associated with geographic distance from a nephrologist. We aimed to determine if differential management of catheter-related blood stream infections (CRBSIs) could explain poorer outcomes.

**Methods:**

We prospectively collected data from adults initiating hemodialysis with a central venous catheter between 2005 and 2015 in Alberta, Canada. We collected indicators of CRBSI management (timely catheter removal, relapsing bacteremia); frequency of CRBSIs; hospitalizations; predictors of CRBSIs, and bacteremia. We evaluated indicators and infectious episodes as a function of the shortest distance by road to the closest nephrologist’s practice: <50 (referent); 50–99; and ≥100 km.

**Results:**

One thousand one hundred thirty-one participants were followed for a median of 755 days (interquartile range (IQR) 219, 1465) and used dialysis catheters for a median of 565 days (IQR 176, 1288). Compared to the referent group, there was no significant difference in the rate ratio (RR) of CRBSI in the 50–100 and >100 km distance categories: RR 1.63; 95% confidence interval (CI) (0.91, 2.91); RR 0.84 (95% CI 0.44, 1.58); *p* = 0.87, respectively or in bacteremia: RR 1.42; (95% CI 0.83, 2.45); RR 0.79 (95% CI 0.45,1.39) *p* = 0.74, respectively. There were no differences in indicators of appropriate CRBSI management or hospitalizations according to distance. The overall incidence of CRBSIs was low (0.19 per 1000 catheter days) as was the frequency of relapse. Only liver disease was independently associated with CRBSI (RR 2.11; 95% CI 1.15, 3.86).

**Conclusions:**

The frequency and management of CRBSIs did not differ by location; however, event rates were low.

## Background

Across a range of populations, living further away from health services is associated with poorer health outcomes [1–6]. This issue is particularly relevant to the delivery of hemodialysis (HD) care, where many patients live and receive treatment in facilities that are far from a nephrologist. As patients living in remote locations must often travel further to receive health care and are seen less often by nephrologists, this geographic barrier may influence the quality of care that remote-dwellers receive [7, 8]. In our previous work based on a large cohort of Canadian HD patients, we found a direct association between the risk of mortality and distance from the closest nephrologist and this association was strongest for death due to infection [8]. However, in order to identify processes of care that are amenable to intervention, a more detailed evaluation of this finding is required.

Infection is the second most common cause of death among HD patients and is a major cause of hospitalization [9]. The use of tunnelled central venous catheters (CVC) for dialysis vascular access contributes to this HD-related morbidity and mortality. Compared to arteriovenous fistulas, tunneled CVCs are associated with a 15-fold greater risk for bacteremia [10]. The management of CVC-related and CVC-unrelated bacteremia are specified in clinical practice guidelines as is close follow up if dialysis catheter salvage is attempted [11]. However, for remote-dwelling patients, dialysis CVC removal typically requires travel, therefore geographic distance could present barriers to the appropriate management of serious infections. We hypothesized that compared to patients living closer to their nephrologists, management of bacteremia will be less optimal in patients who reside at a greater distance, as reflected by a lower odds of timely dialysis catheter removal following bacteremia and a higher odds of relapsing bacteremia.

## Methods

This prospective cohort study is reported according to the STROBE guidelines [12].

### Participants, setting, and data sources

Participants were recruited between March 2005 and March 2015 from the Northern and Southern Alberta Renal Programs. Incident HD patients were enrolled during their initial treatments at major centers (Edmonton, Red Deer and Calgary respectively) and were followed regardless of where they received dialysis throughout the province. It is standard practice for all HD and access-related complications to be managed by the attending nephrologist at the major center as opposed to the local hospital.

Written informed consent was obtained from participants and research ethics boards of the Universities of Alberta and Calgary approved the study. For the purposes of this study adults (≥18 years old) initiating thrice-weekly hemodialysis using catheter access were eligible for inclusion. Participants were followed for up to ten years from initiation of HD or until death or study end (June 2015), whichever came sooner. Participants were censored while using an arteriovenous fistula or graft, receiving home or nocturnal hemodialysis, peritoneal dialysis, or living with a functioning kidney transplant. We resumed following those participants who returned to in-center HD and who were dialyzing via a CVC.

Study participants were enrolled in the Canadian Kidney Disease Cohort Study. Details of the full cohort protocol have been previously published [13]. Briefly for the purposes of this nested study, we collected demographic variables (age, gender, and ethnicity [white, Indigenous or other], smoking status, employment), comorbidities (atrial fibrillation, myocardial infarction, heart failure, hypertension, peripheral vascular disease, dementia, cerebrovascular disease, diabetes, chronic obstructive pulmonary disease, cancers, liver diseases, chronic obesity, psychiatric illness and substance misuse) and clinical management variables (followed by a nephrologist at least 3 months prior to dialysis initiation, year of dialysis initiation, and type of access). Data were collected via participant interviews, chart reviews, and clinical databases at baseline (HD initiation), month six, and years one and two. Demographics and medical history were ascertained at baseline and updated at each ensuing visit. Vascular access type, dialysis modality, date of bacteremic infection, type of organism, whether it was a common skin contaminant or opportunistic, catheter removal and the date of the removal were tracked throughout follow-up. Information on catheter removal was obtained from a clinical database. Episodes of bacteremia were determined prospectively by liaison with the infection control department and then confirmed by microbiology data. We collected the sources of the infection (catheter-related blood stream infection (CRBSI), likely contaminant, secondary or unknown), whether the participant was hospitalized due to the infection (within 2 weeks of the initial event) and the primary diagnosis of the hospitalization (sepsis/sepsis secondary to CRBSI, endocarditis, osteomyelitis, or other). Data were entered and stored in a custom-built Microsoft Access database. Data specific to bacteremic infections were entered and stored in a REDCap database (projectrecap.org).

### Distance to closest nephrologist’s practice

Postal code locations of nephrologist practices were available from the Canadian Organ Renal Replacement Directory. We calculated the driving distance from the postal code centroid of the participant’s residence to the closest nephrologist’s practice. We determined the geographic coordinates of the centroid for each 6-digit postal code using the Statistics Canada Postal Code Conversion File (http://www.statcan.gc.ca/) then transformed them into a projected coordinate space in ArcGIS 10.2 (https://www.esri.com/en-us/home). A topologically sound route was derived from the DMTI route network and network analyses were performed to determine the closest drive distance between the residence of each patient at the time of dialysis initiation and the practice location of their closest nephrologist as previously described [14]. We categorized driving distance to the closest nephrologists’ into the following categories: < 50 km; 50–99 km; and ≥ 100 km. In a sensitivity analysis, driving distance was modeled as a continuous variable.

### Outcomes

The primary outcomes in this study were dialysis catheter removal following bacteremia and relapse of bacteremia. The catheter removal or exchange was deemed timely if it occurred within 14 days of the first positive blood culture. In a sensitivity analysis, timely removal was defined as catheter removal within 72 h of the first blood culture. Based on published guidelines from the study period, catheter removal is required for all episodes of bacteremia with the exception of coagulase-negative Staphylococci. For bacteremia due to coagulase-negative Staphylococci, the CVC may be retained unless there is clinical deterioration or persistent or relapsing bacteremia [15]. Relapse was defined as a repeat positive blood culture with the same species occurring more than two weeks and less than eight weeks from the initial positive blood culture. Secondary outcomes included the frequency of catheter-related blood stream infections (CRBSI) and hospitalization due to CRBSI. In both renal programs, it is standard practice for HD unit staff to draw blood cultures when an infection is suspected clinically (i.e. fever) in the absence of another obvious source of infection. Two adjudicators reviewed the microbiology to confirm which were confirmed CRBSI, possible CRBSI, or secondary sources of bacteremia. A third adjudicator served to resolve any discrepancies. A confirmed CRBSI was defined as one or more blood cultures positive for an opportunistic pathogen (defined a priori) or two or more blood cultures positive for common skin contaminants, drawn within two days of each other. A possible CRBSI was defined as a single blood culture positive for a common skin contaminant. As peripheral blood cultures may not be feasible in HD patients (i.e. due poor peripheral access or saving a vein for future vascular access creation), differential time to positivity was not a requirement for the diagnosis of CRBSI. For episodes of bacteremia not meeting the above criteria, bacteremia from a secondary source was determined based on culture from another source or clinical judgment. Hospitalization due to CRBSI was defined as any hospitalization occurring during the two-week window following the first positive blood culture and any hospitalization with a repeat positive blood culture of the same species within six-months of the first positive blood culture.

### Statistical analyses

All analyses were completed in Stata 13.1 (https://www.stata.com/). Descriptive statistics were reported as counts and percentages, or medians and inter-quartile ranges, as appropriate. To evaluate the association between distance to nephrologist and catheter removal, timely (14-day) catheter removal, and hospitalization (any per time interval), we used unadjusted logistic regression in participants with bacteremic infections; there were too few events to adjust for candidate variables. Relapses were infrequent and not analyzed. To examine the associations between distance to the closest nephrologist’s practice and confirmed or suspected CRBSI and bacteremic infections, we used Poisson regression with a log-link and report adjusted rate ratios. We took a forward stepwise approach to model fitting and forced age and distance to remain in the model. The candidate independent variables are listed in Table 1. All covariates were allowed to vary interview-by-interview. *P* < 0.05 was considered statistically significant.

**Table 1:** Demographics and clinical characteristics of participants at baseline by distance to the nephrologist practice

	< 50 km	50–99 km	≥ 100 km	P
N	896	95	140	
Age, y				0.68
18–49	202 (22.5)	20 (21.1)	30 (21.4)	
50–69	404 (45.1)	41 (43.2)	71 (50.7)	
70+	290 (32.4)	34 (35.8)	39 (27.9)	
Male	541 (60.4)	58 (61.1)	84 (60)	0.99
Ethnicity				< 0.001
White	690 (77)	71 (74.7)	112 (80)	
Indigenous	41 (4.6)	23 (24.2)	22 (15.7)	
Other	165 (18.4)	1 (1.1)	6 (4.3)	
Followed by a nephrologist for ≥ 90 d prior to dialysis start	648 (72.3)	75 (78.9)	92 (65.7)	0.08
Catheter days over full follow-up	567 (181,1315)	565 (168,1260)	563.5 (163,1140)	0.90
Total days of follow-up	754 (223,1456)	1007 (258,1567)	723 (174,1386)	0.48
Morbidities				
Atrial fibrillation	170 (19)	13 (13.7)	21 (15)	0.27
Acute myocardial infarction	201 (22.4)	20 (21.1)	29 (20.7)	0.87
Cancer	111 (12.4)	11 (11.6)	16 (11.4)	0.93
Chronic heart failure	189 (21.1)	21 (22.1)	23 (16.4)	0.42
Chronic lung disease^a^	159 (17.7)	18 (18.9)	26 (18.6)	0.94
Chronic obesity	217 (24.2)	29 (30.5)	52 (37.1)	0.003
Dementia	21 (2.3)	4 (4.2)	1 (.7)	0.21
Diabetes	454 (50.7)	55 (57.9)	86 (61.4)	0.03
Hypertension	783 (87.4)	86 (90.5)	116 (82.9)	0.19
Liver disease	77 (8.6)	7 (7.4)	3 (2.1)	0.03
Peripheral vascular disease	85 (9.5)	8 (8.4)	15 (10.7)	0.83
Psychiatric illness^b^	127 (14.2)	7 (7.4)	20 (14.3)	0.18
Stroke or TIA	128 (14.3)	13 (13.7)	16 (11.4)	0.66
Substance misuse^c^	101 (11.3)	10 (10.5)	19 (13.6)	0.70
Unemployment history^d^	44 (4.9)	10 (10.5)	9 (6.4)	0.07

*IQR* interquartile range, *TIA* transient ischemic attack

N (%) or median (IQR) as appropriate

P for linear trend

^a^Chronic lung disease includes asthma, chronic bronchitis, chronic obstructive pulmonary disease, tuberculosis

^b^Psychiatric illness includes anxiety, depression, bi-polar disorder, and/or schizophrenia

^c^Substances include alcohol, cocaine, ecstasy, intravenous drugs, marijuana, off-prescription drugs, and smoking

^d^Unemployed status includes homemakers and persons receiving income for disability

### Power calculation

A sample size of 1250 participants (approximately 250 collectively in the remote strata and 1000 in the urban stratum) provides >99% power (with a 5% type 1 error rate, a median follow-up of 500 catheter days) to detect an absolute difference in infection rates of 2 infections per 1000 catheter days (assuming a baseline rate of 0.1 infections/1000 catheter days in the urban stratum and 4 infections/1000 catheter days collectively in the remote strata) [16].

## Results

### Participants

**Fig. 1:**
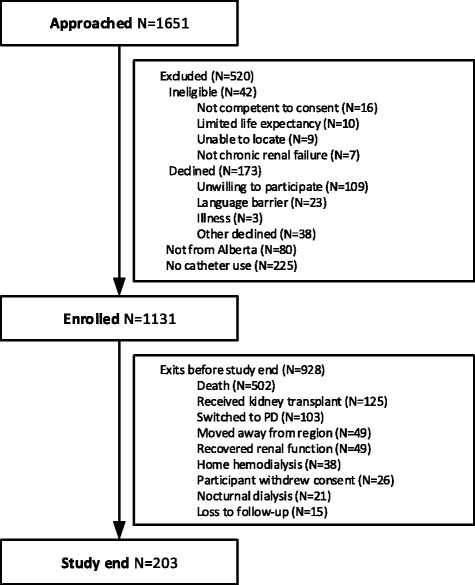
Participant flow diagram. PD peritoneal dialysis

Participant flow is shown in Fig. 1. A total of 1651 HD patients were approached. Of the 520 patients that were excluded, the most common reason was no dialysis CVC in use (*n* = 225) and a lack of interest (*n* = 109). We enrolled 1131 participants over the study period. Participants were followed for a median of 755 days interquartile range (IQR) 219, 1465 and dialyzed via catheters for a median of 565 days (IQR 176, 1288). Characteristics of the 1131 participants were mostly similar across distance categories (Table 1). The prevalence of chronic obesity, diabetes, and Indigenous peoples all increased with increasing distance from a nephrologist practice. The prevalence of liver disease decreased with distance from a nephrologist practice.

### CRBSI and bacteremia

Among the 1131 participants, there were 264 cases of bacteremia, including 164 infections due to confirmed or possible CRBSI (62% of all bacteremia cases). The overall incidence of confirmed or possible CRBSI was 0.19 per 1000 catheter days.

Compared with the referent group (<50 km), there was no association between distance to the closest nephrologist’s practice and the rate ratio (RR) of confirmed or possible CRBSI: RR 1.63; 95% confidence interval (CI) (0.91, 2.91); RR 0.84 (95% CI 0.44, 1.58); *p* = 0.87 for distances of 50–99 km and ≥100 km respectively (Table 2). Similarly, there was no difference in the RR of bacteremic infections: RR 1.42; (95% CI 0.83, 2.45); RR 0.79 (95% CI 0.45,1.39) *p* = 0.74 for those living 50–99 km and ≥100 km away, respectively. The lack of association persisted when distance was modeled as a continuous variable: RR of CRSBI 0.99 (95% CI 0.88, 1.11); *p* = 0.87 and RR of bacteremia 0.98 (95% CI 0.87, 1.09); *p* = 0.74.

**Table 2:** Associations between bacteremia and distance to the nephrologist practice

	Rate ratio of bacteremic infection	Rate ratio of CRBSI
N	1131	1131
Events	264	164
Distance to nephrologist, km		
< 50	1.00	1.00
50–99	1.42 (0.83, 2.45)	1.63 (0.91, 2.91)
≥ 100	0.79 (0.45, 1.39)	0.84 (0.44,1.58)
P for linear trend	0.74	0.87
Age, y		
18–49	1.00	1.00
50–69	0.69 (0.45, 1.05)	0.66 (0.42,1.04)
70+	0.60 (0.38, 0.95)	0.57 (0.35, 0.94)
Morbidities		
Substance misuse^a^	2.32 (1.40, 3.83)	–
Liver disease^b^	1.98 (1.20, 3.27)	2.11 (1.15, 3.86)
Peripheral vascular disease	1.70 (1.11, 2.59)	–
Hypertension	1.37 (1.04, 1.82)	–

CRBSI catheter-related blood stream infection

Fit using forwards stepwise selection using all variables listed in Table 1 and forcing age and distance to closest nephrology practice to remain in the model

^a^Substances include alcohol, cocaine, ecstasy, intravenous drugs, marijuana, off-prescription drugs, and smoking

Liver disease includes alpha-1 antitrypsin disease of the liver, cirrhosis due to any cause, fatty

^b^Liver disease if confirmed on liver biopsy (ever), glycogen storage diseases, hemochromatosis, primary biliary cirrhosis, primary sclerosing cholangitis, veno-occlusive liver disease, and Wilson’s disease

### Catheter removal and hospitalization

Of 132 participants with at least one episode of CRBSI, there were 105 hospitalizations (in 103 participants). Of the 164 infections due to confirmed or suspected CRBSI, the CVC was removed in 135 cases, of which 120 were considered timely catheter removals. We found no evidence that these events were associated with distance from the nephrologist practice: tests for association between linear distance and the odds of dialysis CVC removal, timely catheter removal, and any hospitalization were all non-significant (all *p* > 0.53). When distance from the nephrologist practice was treated as a categorical exposure, the lack of association remained: unadjusted odds ratios for the risk of each outcome were all non-significant for ≥50 km versus <50 km (OR 1.67 (95% CI 0.53, 5.21), 1.19 (95% CI 0.49, 2.90), and 0.93 (95% CI 0.24, 3.56), respectively). When timely catheter removal was defined as 72 h, the lack of association with linear distance remained: OR 0.99 (95% CI 0.98, 1.00); *p* = 0.059. Furthermore, we did not find an association between the odds of catheter removal and microbiology. The odds of catheter removal within 14 days for *Staphylococcus aureus* versus coagulase-negative Staphylococcus was 2.35 (95% CI 0.81, 6.86); *p* = 0.12. There was a trend toward a higher odds of catheter removal within 72 h for *Staphylococcus aureus* versus coagulase-negative Staphylococcus that did not reach statistical significance: 3.83 (95% CI 0.98, 14.98); *p* = 0.054. When bacteremia due to coagulase-negative staphylococci was omitted, there was no relation between linear distance to the nephrologist and the odds of CVC removal at 14 days: 1.00 (95% CI 0.99, 1.01); *p* = 0.56 or at 72 h: 0.99 (95% CI 0.98, 1.00); *p* = 0.08.

### Factors associated with rate of bacteremia

A history of substance misuse, chronic liver disease, peripheral vascular disease and hypertension were all significantly associated with the rate of bacteremia: rate ratio (RR) 2.32 (95% CI 1.40, 3.83), 1.98 (95% CI 1.20, 3.27), 1.70 (95% CI 1.11, 2.59), and 1.38 (95% CI 1.04, 1.82), respectively. Only chronic liver disease was associated with the rate of CRBSI: RR 2.11 (95% CI 1.15, 3.86).

## Discussion

Inequities in health outcomes and quality of care indicators according to remote location have been reported across a number of dialysis cohorts and geographical areas [6, 7, 14, 17, 18]. However, to our knowledge, this is the first report of CRBSI incidence and management according to geographic location. This information is important, as vascular access type and remote residence location are potentially modifiable determinants of poor health outcomes in people requiring HD.

Contrary to our initial hypothesis, we did not find that remote location was associated with a lower odds of timely dialysis CVC removal or a higher frequency of relapsing bacteremia [8]. The overall incidence of CRBSI or secondary sources of bacteremia among remote-dwellers did not differ from that in participants who lived closer. We also found that although morbidity was a risk factor for bacteremia, only liver disease was associated with an increased risk of CRBSI.

There are several potential reasons why we did not find differences in indicators of appropriate CRBSI management or CRBSI rates in remote dwellers versus those who lived closer to a nephrologist. First, the incidence of CRBSI in our cohort (0.19 per 1000 patient days) was considerably lower than reported in other hemodialysis cohorts (1.6–5.5 per 1000 catheter days) [19–21] and statistical power was too low to detect a difference between distance categories. This lower rate of CRBSI was despite the increased prevalence of catheter use in both renal programs over the study period (data not shown). Clinical uptake of interventions with demonstrated efficacy in preventing CRBSI [22] could explain the difference between the rate in our relatively contemporary cohort and previous studies; the routine use of topical antibiotics to the catheter site was implemented in the Northern Alberta Renal Program in 2004. Our findings are consistent with administrative data from the Alberta health authority, which showed a declining rate of infection over the study period from an approximate rate of 0.8 to 0.3 infections per 1000 dialysis runs. Second, in our previous study, [8] an excess risk of infection-related mortality was observed among patients living more than 300 km away from the closest nephrologist and it is possible that the exact location of the majority of participants in the furthest distance category (≥100 km) in the current study was not far enough to impose a barrier to care. Third, residual confounding from unmeasured patient characteristics and regional differences in care delivery and local practices may have also lead to incongruent findings. In one surveillance study of 11 Canadian HD units, substantial variation in infection rates was reported (0.0–2.01 per 1000 catheter days) [16]. In another study, there were substantial between-facility differences in the risk of CRBSI, even after adjustment for potential confounders [23].

Despite the efficacy of interventions to reduce CRBSI, there are likely unrecognized groups who remain at higher risk. Although liver disease leads to an acquired state of immune deficiency, we are not aware that the relation between liver disease and CRBSI has been previously reported. Additional research is required to determine if strategies to optimize vascular access or the routine implementation of interventions aimed at decreasing the incidence of CRBSI could improve clinical outcomes in this group of HD patients. Furthermore, with the exception of access type and a previous episode of bacteremia, many studies evaluating risk factors for CRBSI have reported inconsistent findings and been of limited quality [24]. To facilitate clinical decision-making with specific patient groups on vascular access selection, future studies should prospectively evaluate predictors of CRBSI using multilevel data.

This study has several strengths and limitations that warrant further discussion. First, the prospective design ensured minimal loss to follow-up and accurate capture of infectious events. Second, in contrast to previous studies, we included coagulase-negative Staphylococcus isolates as the majority of these findings likely represent true bacteremia rather than contamination [23]. For cases of coagulase-negative Staphylococcus, we did not collect data on the indication for CVC removal. Although differences in CVC removal according to microbiology were not detected, it would be important for future studies with higher rates of CRBSI to examine whether conservative management with antibiotic locks for specified microorganisms is associated with poorer outcomes for remote-dwelling HD patients. Third, we did not include sampling from a peripheral vein (as recommended by the Centres of Disease Control and Prevention) [11]. Given the challenges in diagnosing CRBSIs in this population (i.e. poor peripheral venous access, preserving veins for fistula and uncertainty of the value of differential time to positivity when cultures are drawn after the systemic blood has circulated through the catheter), we used a definition of CRBSI that is appropriate for outpatient HD practice. Our chosen definition is also valid, Pelletier et al. [25] showed that compared with blood cultures from the dialysis circuit and the catheter hub, an additional culture from the peripheral vein did not improve the accuracy, sensitivity, or specificity of CRBSI diagnosis. In addition, to minimize error in misdiagnosing CRBSI, we used an adjudication process with clearly defined criteria. Fourth, as patients could have moved during the study period, we modeled location as time varying covariate. Fifth, our cohort only included patients treated with a CVC and it is possible that infection-related deaths in remote-dwelling HD patients may be due to non-CRBSIs. However, if death from infection were independent of access type, we would have observed differences in the rate of bacteremia. Sixth, we did not collect information on the timing and type of antibiotic; however, relapsing bacteremia is a more informative outcome that would also capture treatment delays. Finally, and perhaps most importantly, this study did not include an analysis of cause-specific mortality.

## Conclusions

In this prospective cohort study of HD patients, we found that distance from the closest nephrologist did not influence indicators of appropriate CRBSI management, or the rate of CRBSI or bacteremia. However, given that the relation between remote location and poorer health outcomes has been consistently shown across numerous populations and that our negative findings were influenced by a low CRBSI rate, additional studies are required.

## Abbreviations

CRBSI: Catheter-related blood stream infection
CVC: Central venous catheter
HD: Hemodialysis
